# Pharmacological Agent GW4869 Inhibits Tick-Borne Langat Virus Replication to Affect Extracellular Vesicles Secretion

**DOI:** 10.3390/v17070969

**Published:** 2025-07-10

**Authors:** Md Bayzid, Biswajit Bhowmick, Waqas Ahmed, Girish Neelakanta, Hameeda Sultana

**Affiliations:** Department of Biomedical and Diagnostic Sciences, College of Veterinary Medicine, University of Tennessee, Knoxville, TN 37996, USA

**Keywords:** GW4869, tick cells, Langat virus, EVs secretion, cytotoxicity, viral dilution, viral replication, viral particle infectivity

## Abstract

GW4869, a cell-permeable, selective inhibitor of neutral sphingomyelinase is a pharmacological agent that blocks the production and release of extracellular vesicles (EVs). Our previous studies have shown that GW4869 inhibits flaviviral loads in tick, mosquito and mammalian cells, including murine cortical neurons. Yet the mechanism(s) of GW4869 inhibitor upon viral infections were not addressed. In the current study, we focused on how GW4869 interferes with Langat Virus (LGTV, a tick-borne flavivirus) replication in ISE6 tick cells. First, we found that GW4869 is neither cytotoxic at tested doses of 50, 100, and 150 µM in tick cells, nor does it directly bind to the free LGTV present in cell culture supernatants. When tick cells were treated with GW4869, followed by infection with viral stock at dilutions of 10^−2^, 10^−3^, 10^−4^ (the infectious dose determination by viral dilution assay), it affected LGTV replication in tick cells. A reduction in viral burden was noted in GW4869-treated tick cells, which constituted more than half the amount of decrease when compared to the mock control. Next, GW4869 treatment not only resulted in decreased LGTV transcript levels in tick cells and EVs derived from these infected cells, but also revealed diminished EVs concentrations. Enhanced *Is*SMase transcripts in the LGTV-infected group was noted upon GW4869 treatment, thus suggesting a host response to perhaps inhibit virus replication. In addition, GW4869 treatment reduced LGTV loads in density gradient EVs fractions, which correlated with decreased EVs concentration in those fractions. These data not only indicate that GW4869 affects LGTV replication, but that it also interferes with EV secretion and release from tick cells. Lastly, we found that GW4869 inhibits LGTV replication in tick cells but does not directly affect the infectivity of LGTV viral particles. Overall, our study suggests that GW4869 is a potential therapeutic inhibitor in controlling tick-borne diseases.

## 1. Introduction

The dynamics of extracellular vesicle (EV) internalization, or fusion to recipient cells, their biogenesis and trafficking, rely heavily on the physiological and pathological conditions of those recipient cells [1,2,3,4,5,6,7,8,9]. The production and release of EVs from recipient cells could be irregular and unbalanced during viral infections [2,4,6,7,8,9]. The cargo sorting mechanism(s) in those infectious cells are perhaps, far different than those of the normal healthy cells [10,11]. To explore and advance the mechanisms of EV–cell interactions and their biogenesis, some inhibitors or blocking substances have been developed in the field [1,2,3,4,5,6,7,8,9]. One such potential inhibitor is GW4869, which is a cell permeable, selective inhibitor of neutral sphingomyelinase (N-SMase2/SMPD3) that blocks the production and release of EVs in treated cells [6,7,8,9]. In our previous study, we have shown that treatment of murine cortical neurons with GW4869 at low doses of 5, 10 and 20 µM significantly reduced ZIKA virus levels (ZIKV is a mosquito-borne flavivirus) at 24, 48 and 72 h post-infection [9]. Also, ZIKV-Envelope (E) protein levels were drastically reduced upon GW4869 treatment (in a dose-dependent manner at 24, 48 and 72 h post viral infection) [9]. In addition, both ZIKV transcripts and E-protein levels were reduced in EVs derived from GW4869-treated murine cortical neurons [9]. The inhibition of ZIKV loads correlated with significantly reduced SMPD3 transcript levels in both cortical neuronal cells and in infectious EVs derived from these cells at the tested low doses of 5, 10 and 20 µM and at timepoints of 24, 48 and 72 h post-infection [9].

Mosquitoes and ticks are important vectors and transmit several human and animal pathogens of high priority, such as flaviviruses [4,7,8,12,13,14,15,16,17,18,19]. Our previous studies have shown that the tick-borne Langat virus (LGTV), a flavivirus closely related to tick-borne encephalitis virus (TBEV), Powassan virus (POWV) and other family members, readily infect the *Ixodes scapularis* tick cell line (ISE6) [4,8]. We detected increased viral RNA levels at 72 h post LGTV-infection. Also, we noted enhanced release of EVs at this tested timepoint of 72 h post LGTV-infection [4,8]. We manually counted EVs and found higher numbers from LGTV-infected (72 h p.i.) tick cells in comparison to EVs derived from uninfected tick cells [8]. Tick cell derived EVs were heterogenous in population and were of variable sizes (from 50–350 nm); however, they were mostly of 50–100 nm in diameter [8]. It was noted that LGTV-infected tick cell-derived EVs were infectious, as they efficiently transmitted the viral RNA levels to naïve recipient mammalian cells like human-skin keratinocytes (HaCaT cells) or blood endothelial cells (HuVEC cells) [8]. In the presence of GW4869 inhibitor (at low tested doses of 1 µM for tick cells or 1, 5 or 10 µM for mouse neuronal N2a cells), LGTV transmission via infected tick cell/neuronal cell-derived EVs to the respective HaCaT cells or bEND.3 (brain microvascular endothelial) cells was significantly reduced [8]. Pre/post treatment of mouse N2a cells with GW4869 (at 5 µM) showed reduced LGTV loads. However, the effect on viral RNA levels from pre-treatment of N2a cells was higher compared to the effects noted from post-treatment of these cells. Our recent work showed that GW4869 treatment of nymphal ticks (either simultaneous or pre-treatment at 150 µM) reduced the LGTV dissemination from midguts to salivary glands (SGs) and other tissues within infected ticks [6]. This reduction in viral dissemination from midguts to SGs was also noted in fed nymphal ticks, which naturally acquired the LGTV burden from the infected murine host [6]. The reduced viral RNA levels in SGs directly correlated with decreased EV concentration in molted adult tick SGs [6]. In the current study, we investigated LGTV loads in tick cells and EVs derived from these infected cells upon GW4869 treatment, and measured EV concentration. Also, we analyzed the direct effects of GW4869 on free viruses in suspensions and in the viral replication. We addressed how GW4869 affects LGTV replication and interferes with EV release in tick cells. We also tested whether GW4869 treatment affects the ability of LGTV viral-particle infectivity in naïve tick cells. Our study highlights how GW4869 interferes with Langat virus replication in ISE6 tick cells.

## 2. Materials and Methods

**Cell culture, GW4869 treatment and infection of ISE6 tick cells.** *Ixodes scapularis* (ISE6) tick cell line (obtained from BEI resources, USA; catalog number NR-12234) was used in all in vitro experiments and maintained as described [4,8,20]. Laboratory stocks of wild-type Langat virus (LGTV, strain LGT-TP21, obtained from BEI resources and propagated in Vero cells) was used in this study. Briefly, 5 × 10^5^ tick cells resuspended in L15B300 complete cell-culture media with 5% FBS (fetal bovine serum, VWR, USA) were seeded in a 12-well plate. The L15B300 media was prepared in the laboratory, as per recommendations from Dr. Munderloh [21,22]. After overnight incubation, the cell culture media was replaced with fresh media containing 5% FBS (Systems Biosciences Innovation, SBI) depleted with bovine EVs. After 4 h incubation with Exo-free FBS, tick cells were treated (for 4 h) with 1 or 150 µM of GW4869 inhibitor (Santa Cruz Biotechnologies, Inc., Dallas, TX, USA) or with 0.01% or 1.5% DMSO (as mock), respectively. GW4869 is prepared in DMSO solution (obtained from SIGMA, St. Louis, MO, USA) and was used as control in the study. Following the incubations, the tick cells were infected with LGTV (with one multiplicity of infection, MOI 1) in the same media containing GW4869 inhibitor [4,8]. After 3 days post-LGTV infection (p.i.), tick cell culture supernatants were harvested and processed immediately for EV isolation by ultracentrifugation or density-gradient ultracentrifugation methods, as described in our previous studies [4,8,9,19,20]. Tick cells or purified EVs were collected in RLT (RNA extraction) or RIPA (protein extraction) lysis buffers and processed further [4,6,8,20], as per the instructions from the companies Bio-Rad Laboratories, Hercules, CA, USA, or Thermo Fisher Scientific, Waltham, MA, USA, respectively. For direct treatment of LGTV virus stocks (as undiluted virus stock with titer of 1 × 10^8^ pfu/mL or diluted virus as MOI 1), 100 µL of undiluted (1 × 10^8^ pfu/mL) or diluted (1 × 10^5^ pfu/mL, as 1 MOI) viruses was aliquoted into sterile tubes. We considered MOI 1, because 1 × 10^5^ density of tick cells were plated for all experiments shown in this study. These virus suspensions (6 replicates for each group) were treated with 1 µM of GW4869 (for 4 h at 37 °C). In addition, the LGTV viral stock (of known titers—1 × 10^7^ pfu/mL) was either treated with mock (DMSO, 0.01%) or with GW4869 (1 µM, for 4 h at 37 °C), followed by RNaseA treatment (5 μg/mL, for 15 min, at 37 °C) (RNaseA obtained from SIGMA, USA). All samples were collected for viral RNA/protein extractions.

**LDH-Glo Cytotoxicity Assay.** The ISE6 tick cells (5 × 10^5^) were plated in L15B300 complete media. After overnight incubation, we replaced the complete media with Exo-free FBS media (as described above) for 4 h. Uninfected tick cells were treated with GW4869 inhibitor (at doses of 0–150 µM, for 4 h). The mock control group, with no infection, was also treated with 1.5% of DMSO (which matches the highest tested dose of 150 µM treatment of GW4869). Each dose and mock control were tested as 6 independent replicates and in duplicate. After 3 days post incubation with either GW4869 or mock treatments, the tick cells were carefully observed for any cytotoxic effects and imaged for assessing tick-cell morphology. The tick cells were processed for the LDH-Glo cytotoxicity assay [23], which is well suited for measuring the LDH release from membrane-damaged cells, following the instructions from the manufacturer (Promega, Madison, WI, USA). The luciferase readouts are shown as emitted light in nm. The light signal generated is proportional to the amount of LDH release that is reflected as related luminescence.

**MTT assay.** Briefly, the tick cells were seeded (in a 96-well plate) at a density of 5 × 10^4^ cells/well in 225 µL of L15B300 complete tick-cell culture media. After overnight incubation, cells were treated with mock (1.5% DMSO) or with GW4869 inhibitor (at two tested doses of 50 or 150 µM for 4 h) followed by additional incubation. After 72 h post incubation, we added 22.5 µL of MTT solution [9]. The plate was wrapped with aluminum foil and incubated at 37 °C for 3 h, and thereafter, 100 µL DMSO was added to each well, respectively. The plates were incubated for another 15 min at 37 °C and absorbance was determined at optical density of 570 nm and 690 nm. Each group had 8 replicates that were run as duplicates. Differences in values determine the cell viability numbers with the higher absorbances indicating increased viability of the tick cells.

**End-point viral dilution assay.** We performed the viral dilution assay as described in [7], to determine the tissue culture infectious dose (TCID_50_) for the virus titers. Briefly, the tick cells were seeded (at densities of 1 or 5 × 10^4^ cells/well of a 96-well plate) in 225 µL of L15B300 complete medium. After overnight post-plating, the tick cells were replaced with Exo-free FBS media and treated with GW4869 inhibitor (with 1 or 150 µM, for 4 h) or mock control (DMSO with 0.01% for 1 µM or 1.5% for 150 µM treatments, respectively), followed by infection with LGTV (with laboratory viral stock, which is serially diluted from 10^−1^ to 10^−6^) and incubated for an additional 3 or 6 days. For each dilution group, at least 6 independent replicates were included, in addition to the uninfected group that served as the internal control. Images from dilutions of 10^−2^ to 10^−4^ are shown from both the mock and the GW4869-treated groups, and for two different doses of GW4869 (1 and 150 µM). The tick cells were fixed with acetone-PBS mixture (in 3:1 ratio, for 20 min at −20 °C) and plates were air dried, washed with 1 × PBS and blocked with 5% FBS and PBS-solution with 0.05% sodium azide for 15 min, at room temperature (RT). LGTV-Envelope (E)-protein was detected by incubation (overnight at 4 °C) with 4G2 monoclonal antibody (catalog number NR 50327, obtained from BEI Resources, Manassas, VA, USA), followed by three washes with 1 × PBS. Samples were incubated with Alexa-594-labeled mouse secondary antibody (obtained from Thermo Fisher Scientific, Inc., USA) for 1 h, at RT, followed by washes (3×) with 1 × PBS. After secondary antibody incubation, the cells were counter-stained with DAPI (0.5 µg/mL for 3 min, obtained from Thermo Fisher Scientific, Inc., USA). The plates were analyzed using Cytation7 imaging system (Bio-Tek/Agilent, Santa Clara, CA, USA). The tick cells were scored for fluorescence or presence of infection in comparison to the uninfected control (used as negative control for LGTV infection). TCID50 values are converted to pfu/mL to obtain the virus titers. Representative images from dilutions of 10^2^–10^4^ (at day 3 or 6 p.i.) are shown. Images are obtained at 20X magnification. The scale bar (200 µm) is shown on each representative image from the respective groups.

**Isolation and nano-quantification of EVs derived from tick cells.** Tick cells (plated at densities of 5 × 10^5^ cells per well and as 5–6 replicates, for collection of total RNA) were treated for 4 h with either mock (0.01% or 1.5% DMSO) or GW4869 (1 or 150 µM) inhibitor, followed by LGTV infection (MOI 1 for 3 days p.i.). Tick-cell culture supernatants were individually (per well) processed for EV isolation (by using ultracentrifugation or density-gradient methods for collection of Evs or fractions), as described [4,8,20]. For protein extractions, the tick cells were plated at 1–2 × 10^6^ densities in 6-well plates and processed for EV isolation using the density-gradient ultracentrifugation method, as described in [4,8,20]. Pelleted Evs were collected in cold 1 × PBS, and as multiple replicates for each group. Isolated Evs (in 1 × PBS) were briefly stored or quantified immediately with nCS1 nanoparticle analyzer (Spectradyne Particle Analysis, Signal Hill, CA, USA), following the manufacturer’s recommendations or as described in [20]. Isolated EVs from tick-cell culture supernatants were diluted (1:2000) in 1% Tween-20 prepared in filtered 1 × PBS. Diluted samples (5 µL) were loaded onto nCS1 microfluidic cartridge TS-400 with a size range of 65–400 nm (in diameters). Loaded cartridges were inserted in nCS1 analyzer to quantify EVs from each sample. Data was collected from four independent replicates and processed as described in [20].

**RNA extraction, cDNA synthesis, and RT-qPCR analysis.** Total RNA was extracted from GW4869-treated (1 or 150 µM, for 4 h) or mock-treated (0.01% or 1.5% DMSO) tick cells. RNA was extracted using the Aurum Total RNA Mini Kit (Bio-Rad, USA), following the manufacturer’s instructions. Viral RNA was extracted using a QIAamp viral RNA mini kit (Qiagen, Germantown, MD, USA). Extracted RNA (1 μg) was converted using an iScript cDNA Synthesis Kit (Bio-Rad Laboratories, USA). Total RNA was processed for cDNA synthesis and quantitative reverse-transcription polymerase chain reaction (RT-qPCR) analysis [4,8]. RT-qPCR reactions were performed using SYBR Green Supermix and a CFX-OPUS instrument (Bio-Rad Laboratories, USA) [24]. Published gene-specific primers were used to detect LGTV RNA [4,8], *Ixodes scapularis* Sphingomyelinase (*Is*SMase) transcripts, and tick beta-actin levels [4,8]. LGTV loads were normalized to total tick-cell RNA or viral RNA, respectively, whereas *Is*SMase transcript levels were normalized to tick beta-actin levels. Gene fragments were amplified, and cDNA concentrations were determined for the first standard. Standard curves were prepared for each gene using 10-fold serially diluted standards ranging from 1 to 0.00001 ng/μL of known quantities of the respective fragments.

**Immunoblotting Analysis.** Western blotting was performed, as previously described [7,8]. Briefly, 1–2 × 10^6^ tick cells were seeded in 6-well plates for overnight incubations. The tick cells were treated with GW4869 inhibitor (at 1 μM for 4 h), followed by LGTV infection (MOI 1 for 72 h p.i.), and EVs were isolated as I-VI fractions (by the density-gradient ultracentrifugation method) [7,8,20]. Total protein lysates were collected from EVs (as I–VI fractions) and resuspended in modified RIPA lysis buffer. The Bradford assay (BCA kit from Pierce™ Bradford Protein Assay Kit, Thermo Fisher Scientific, USA) was performed to determine the total protein amounts from EV fractions, and these were processed for immunoblotting analysis. Total lysates prepared from EVs (22 μg from each fraction, I–VI) were separated onto 12% SDS-PAGE gels (reducing conditions). Total protein-profile gel images served as loading controls. After gel electrophoresis, the total proteins were transferred onto nitrocellulose membranes, which were later blocked overnight in 5% milk and 1 X TBST buffer (at 4 °C, a Tris-buffered saline solution containing Tween 20 detergent) and probed with mouse monoclonal antibody against Langat virus Nonstructural protein 1 (NS1) (clone 6E11, catalog number, NR-40308; obtained from BEI Resources, USA) with dilutions of 1:1000 (overnight, at 4 °C). After washes, the blots were incubated with HRP-conjugated mouse secondary antibody (at 1:5000 dilutions, at RT for 1 h). Blots were developed by incubating with ECL reagents (Clarity™ Western ECL Substrate Kit, VWR, Radnor, PA, USA). For blot documentation and analysis, the Chemidoc MP imaging system with Image Lab Touch Software and Version 2.4.0.03 (Bio-Rad Laboratories, USA) was used, according to the manufacturer’s recommendations. The densitometry analysis shown, for comparison of viral NS1 protein in fractions obtained from mock and GW4869-treated tick cells, was performed using ImageLab software.

**Viral particle infectivity Assay.** To determine the effects of GW4869 treatment on viral particle infectivity, we collected our laboratory viral stock of LGTV (with infectious titers of 1 x 10^9^ pfu/mL). We diluted this stock to 10^8^ pfu/mL with complete tick-cell culture media, and aliquoted it as 50 µL vials (12 replicates of 50 µL each). We treated 6 of the 50 µL vials with mock (1.5 % of DMSO as the vehicle, for 4 h) and 6 other vials of 50 µL were treated with GW4869 (150 μM, for 4 h). We plated naïve/uninfected tick cells (at a density of 5 x 10^5^ cells/per well in a 12-well plate, overnight) and the next day we infected 6 wells with 5 µL of mock-treated LGTV and another 6 wells with 5 µL of GW4869-treated virus. These tick cells were further incubated for either 24 h or 72 h, post LGTV infection. The tick cells were then collected for total RNA extraction, followed by cDNA synthesis and RT-qPCR analysis, to determine the viral loads.

**Statistical Analysis.** GraphPad Prism 6 or 9 software (https://www.graphpad.com/) and Microsoft Excel 2010 (https://www.microsoft.com) were used to analyze all data collected in this study. Statistical analysis was performed by a non-paired, two-tail Student’s *t*-test or two-tail *t*-test with Welch’s correction analysis for comparison of the two groups, or two-way ANOVA analysis (with Greenhouse–Geisser correction) for multiple group comparison. Error bars represent mean (+SD) values, and *p*-value < 0.05 was considered statistically significant in all analyses.

## 3. Results

**Figure 1:**
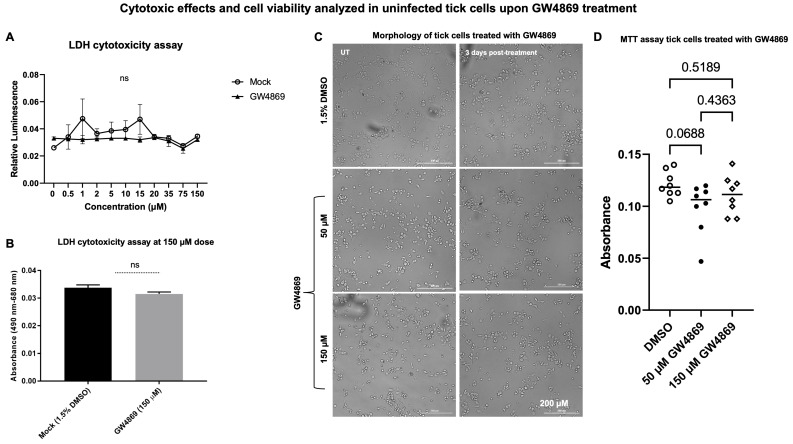
LDH-Glo cytotoxicity and MTT assays performed in tick cells treated with GW4869. (**A**) Uninfected tick cells treated with GW4869 (at 0–150 µM doses) or mock (1.5% DMSO solution), showing LDH release measured by absorbance. (**B**) Cytotoxicity measurement at 150 µM of GW4869 treatment is shown independently, as a bar graph, in comparison to the 1.5% DMSO mock control. In both panels, (**A**,**B**), ns indicates not significant. (**C**) Microscopic images showing morphology of uninfected tick cells treated with GW4869 (at 50 or 150 µM doses), or mock control (1.5% DMSO) from untreated (UT), or 3 days post-GW4869-treatment groups. Tick cell images were obtained using the Cytation7 imager. Scale bar indicates 200 µm. (**D**) MTT assay performed on uninfected tick cells treated with GW4869 (at 50, or 150 µM doses) or mock control (1.5% DMSO) is shown. Each circle (open/close) or rhombus represents one experimental replicate. A sample size of n = 6 was used, with each replicate run in duplicate (in panels (**A**,**B**,**D**)). The *p*-value less than 0.05 is considered statistically significant.

**GW4869 treatment has no cytotoxic effects in tick cells.** Our previous studies [4,8] showed that LGTV infection enhanced EV release in tick cells, but GW4869 treatment (at 1 µM) reduced viral RNA levels and, perhaps, replication efficiency of LGTV in tick cells. The effects of GW4869 treatment on the release of EVs from tick cells upon LGTV infection were not investigated, and we address this in the current study. First, we performed a LDH cytotoxicity assay in uninfected tick cells treated with either mock (1.5% DMSO) or GW4869 inhibitor (at different tested doses of 0–150 µM, for 3 days post treatment). No significant differences were noted between mock or GW4869-treated groups (at 150 µM, as the highest tested dose), nor with lower doses of GW4869 treatment (Figure 1A,B). No cytopathic effects or changes in cell morphology were noted for GW4869-treated tick cells (at both 50 and 150 µM) in comparison to the mock-treated control group (Figure 1C). Tick cells are very small in diameter, and hence higher magnification insets are shown (Supplementary Figure S1). In addition, we performed an MTT assay to determine tick-cell viability upon GW4869 treatment (at both 50 and 150 µM tested doses). No significant differences were noted for tick cell viability between the mock and GW4869-treated groups (Figure 1D). These data show that GW4869 treatment has no cytotoxic effects, nor does it affect the viability of tick cells.

**Figure 2:**
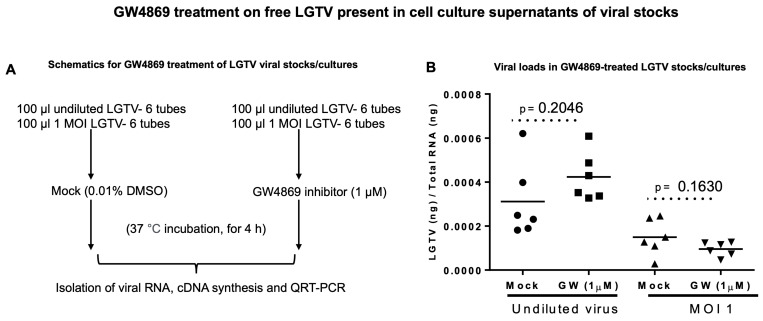
GW4869 effects on free LGTV present in laboratory viral stock suspensions. (**A**) Schematics showing experimental plan for the incubation of LGTV laboratory viral stocks (undiluted suspension with 1 × 10^8^ pfu/mL or diluted as MOI 1) with GW4869 (1 µM) or with mock (0.01% DMSO) control (for 4 h at 37 °C incubation), followed by isolation of viral RNA. (**B**) RT-qPCR analysis showing LGTV viral loads in mock/GW4869-treated undiluted or diluted virus stocks. LGTV prM transcript levels were normalized to total viral RNA levels. Solid circles/triangles denote the mock-treated groups, whereas the squares/inverted triangles represent the GW4869-treated groups. Each circle/triangle/square/inverted triangle represents one experimental replicate (100 µL) of the respective virus suspension. The *p*-value less than 0.05 is considered statistically significant.

**GW4869 does not affect LGTV RNA.** To understand the direct effects of GW4869 on LGTV viral RNA content, we considered incubating the undiluted laboratory virus stocks (of known titer 1 × 10^8^ pfu/mL) or a dose of MOI 1 of LGTV (diluted from the laboratory virus stock) with GW4869 (at 1 µM, for 4 h) or with relevant mock control (of 0.01% DMSO). Viral prM transcript level detection by RT-qPCR analyses revealed no significant differences between undiluted virus stocks. We found no differences in the MOI 1 diluted groups treated with GW4869 or the mock control. (Figure 2A,B). Also, we treated the mixtures of undiluted virus stocks (of known titer 1 × 10^7^ pfu/mL, an independent batch of LGTV stock) and GW4869 inhibitor or DMSO control (at 1 µM, for 4 h) with RNaseA (5 μg/mL, for 15 min, at 37 °C). No significant differences were noted between the mock/GW4869-treated groups incubated with RNaseA treatment (Supplementary Figure S2A,B). These data show that GW4869 does not affect LGTV levels in suspensions of laboratory viral stock.

**Figure 3:**
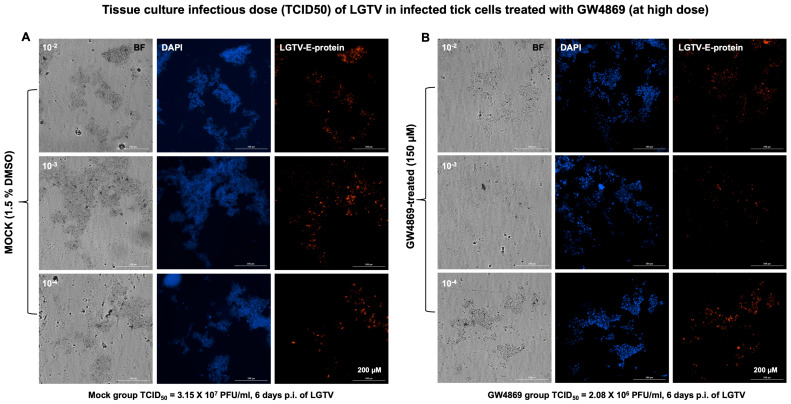
Determination of LGTV infectious dose in GW4869-treated (at 150 µM) tick cells. Bright-field (BF) and fluorescence images collected from viral dilution assay of LGTV-infected tick cells treated with either mock (1.5% DMSO) control (**A**) or with GW4869 (150 µM) treatment (**B**) from dilutions of 10^−2^, 10^−3^ and 10^−4^ are shown. Blue is DAPI staining showing cell nuclei, and red staining detects LGTV envelope (E) protein. Differences in viral titers determined from mock or GW4869-treated tick cells are shown below images as Mock/GW4869 group TCID_50_ values in PFU (plaque-forming units) per ml. Tick cell images were obtained using Cytation7 imager and a scale bar of 200 µm is shown for each image.

**GW4869 affects LGTV infectivity and viral titers in tick cells.** Furthermore, we analyzed the viral infectivity or tissue culture infectious dose (TCID_50_) by virus dilution assays performed on tick cells treated with GW4869 (at 1 or 150 µM, for 4 h) or mock (0.01% or 1.5% DMSO, respectively) control, followed by incubations at different dilutions (10^2^, 10^3^ and 10^4^ are represented in the respective figures) of LGTV prepared from laboratory virus stocks (of known titers of 1 × 10^8^ pfu/mL). We found reduced viral infectivity in GW4869-treated (at both 1 or 150 µM) tick cells when compared to their respective mock-treated control groups and at all tested dilutions (Figure 3A,B for 150 µM and Supplementary Figure S3A,B for 1 µM treatments). GW4869-treated tick cells (at both 1 or 150 µM doses) showed reduced LGTV infectious viral titers (at all tested dilutions of 10^2^, 10^3^ and 10^4^) in comparison with their respective mock-treated tick cells (Figure 3 and Figure S3). Tissue culture infectious dose (TCID_50_) calculations revealed 3.25 × 1e7 pfu/mL (for 150 µM, and 6 days post incubation) or 6.1 × 10^4^ pfu/mL (for 1 µM, and 3 days post incubation) of viral titers for the mock-treated group. We noted that the GW4869-treated group had reduced viral titers of 2.08 × 10^6^ pfu/mL (for 150 µM) or 2.3 × 10^4^ pfu/mL titers (for 1 µM). These data show that GW4869 inhibitor does not directly affect LGTV RNA, but it does interfere with the viral infectivity and titers in tick cells.

**Figure 4:**
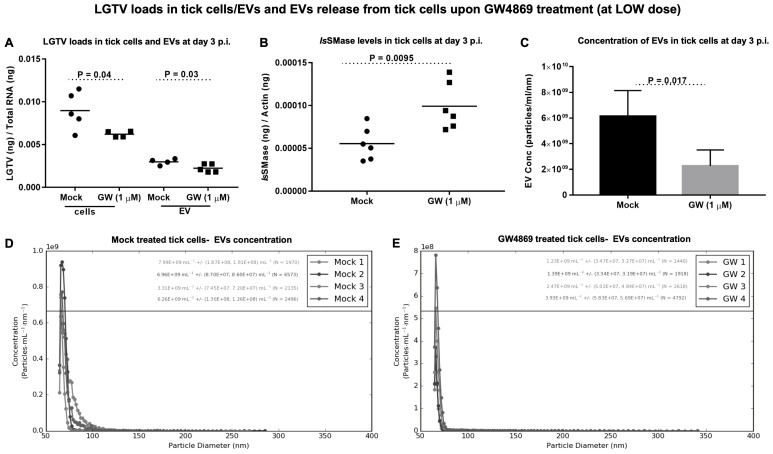
LGTV loads upon GW4869 treatment (low dose, 1 µM) in tick cells/EVs and EV release from tick cells. (**A**) RT-qPCR analysis showing LGTV loads in mock (0.01% DMSO) or GW4869-treated (1 µM, for 4 h) tick cells and EVs derived from these infected tick cells (MOI 1, 3 days post-infection, p.i.). (**B**) *Is*SMase transcript levels in mock/GW4869-treated tick cells infected with LGTV (MOI 1, 3 days p.i.) are shown. (**C**) Concentration of EVs determined by nCS1 analysis from mock/GW4869-treated tick cells infected with LGTV is shown. Graphical representations showing EV quantification by nCS1 analyzer in mock- (**D**) or GW4869-treated (**E**) tick cells infected with LGTV. The diameter of each particle and concentration of EVs was analyzed. Five-six independent replicates in duplicate were considered for RT-qPCR analysis and four independent replicates in duplicate were used for measuring EV concentration. LGTV and *Is*SMase transcripts were normalized with either total RNA or tick beta-actin transcript levels, respectively. Each solid circle denotes the mock-treated group, whereas each closed square represents the GW4869-treated groups (in panels (**A**,**B**)). Both groups were infected with LGTV. Each circle/square denotes data value from one independent culture well. The *p*-value less than 0.05 is considered as statistically significant.

**Figure 5:**
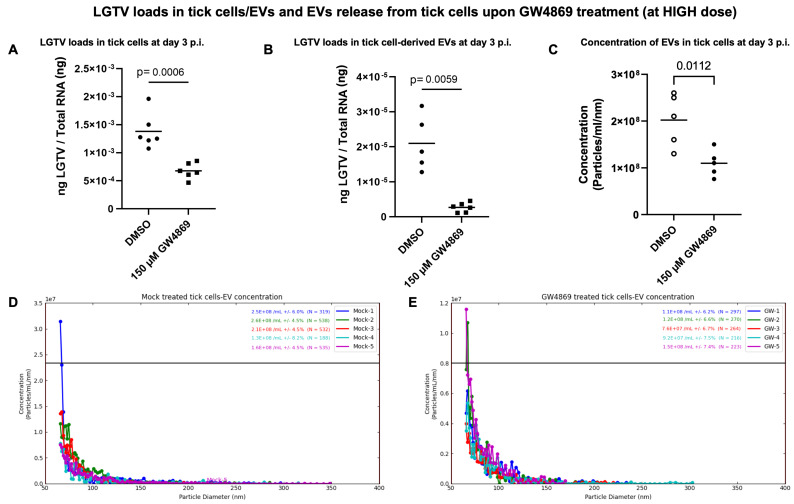
LGTV loads upon GW4869 treatment (high dose, 150 µM) in tick cells/EVs and EV release from tick cells. (**A**) RT-qPCR analysis showing LGTV loads in mock (1.5% DMSO) or GW4869-treated (150 µM, for 4 h) tick cells and (**B**) in EVs derived from these infected tick cells (MOI 1, 3 days post-infection, p.i.). (**C**) Concentration of EVs determined by nCS1 analysis from mock/GW4869-treated (150 µM) tick cells infected with LGTV is shown. Graphical representations showing EV quantification by nCS1 analyzer in mock (**D**) or GW4869- (**E**) treated (150 µM) tick cells infected with LGTV. The diameter of each particle and concentration of EVs was analyzed. Five-six independent replicates in duplicate were considered for RT-qPCR analysis and five independent replicates in duplicate were used for measuring EV concentration. LGTV transcripts were normalized to total RNA levels, respectively. Each solid circles denotes a mock-treated group, whereas each closed square represents the GW4869-treated groups (in panels (**A**,**B**)). Both groups are infected with LGTV. Each circle/square denotes data value from one independent culture well. The *p*-value less than 0.05 is considered as statistically significant.

**GW4869 treatment reduced LGTV loads in ISE6 tick cells and EVs and affects EV secretion.** Tick cells treated with GW4869 (at 1 or 150 µM) or mock (0.01 or 1.5% of DMSO) for 4 h followed by LGTV infection (with MOI 1, for 3 days post-infection (p.i.)) confirmed significantly (*p* < 0.05) reduced LGTV loads when compared to their respective mock-treated controls (Figure 4A and Figure 5A). We also noted that EVs derived from these GW4869-treated (1 or 150 µM doses) LGTV-infected tick cells had significantly reduced viral RNA levels in the GW4869-treated group in comparison to their respective mock-treated control groups (Figure 4A and Figure 5B). In addition to reduced LGTV loads, we further confirmed that *Is*SMase transcript levels were significantly upregulated in GW4869-treated (1 µM) LGTV-infected tick cells in comparison to the mock-treated control group (Figure 4B). These data supported our previous observation [4], which suggests treatment with GW4869 reduces LGTV loads and restores viral-mediated decrease of both *Is*SMase levels and sphingomyelinase activity. Furthermore, to address the direct effects of GW4869 (at both 1 or 150 µM doses) on its ability to reduce LGTV replication in tick cells and derived EVs, we quantified the EV concentrations (diluted with 1 × PBS/Tween 20 (PBST) in a ratio of 1:2000) by using Spectradyne’s nCS1^TM^ particle analyzer. GW4869 treatment (at 1 µM) resulted in more than 50% reduction in EV concentration when compared to the mock-treated control group (Figure 4C). Measurement of EV concentration revealed a lower number (N = 3694 and 2692) and reduced concentration (6.09 × 10^9^ ± (1.20 × 10^8^, 1.16 × 10^8^)/mL and 2.26 × 10^9^ ± (4.41 × 10^7^, 4.24 × 10^7^)/mL of EVs derived from the GW4869-treated (at 1 µM) LGTV-infected group, in comparison to the EVs derived from the mock-treated LGTV-infected control group (Figure 4D,E). Likewise, GW4869 treatment at 150 µM revealed significantly reduced EV concentrations when compared to the respective mock control group (Figure 5C). Measurement of EV concentration (as per the newly updated nCS1 analysis software, nCS1.2.5.0 version) revealed a lower number (N = 422 and 254) and reduced concentration (2.02 × 10^8^ mL (± 5.54%) and 1.10 × 10^8^ mL (±6.88%)) of EVs derived from the GW4869-treated (at 150 µM) LGTV-infected group, in comparison to the EVs derived from the mock-treated LGTV-infected control group (Figure 5D,E). These observations of the reduction in EV number and concentration correlated with reduced viral RNA levels in tick cells and EVs that were noted upon GW4869 treatment.

**Figure 6:**
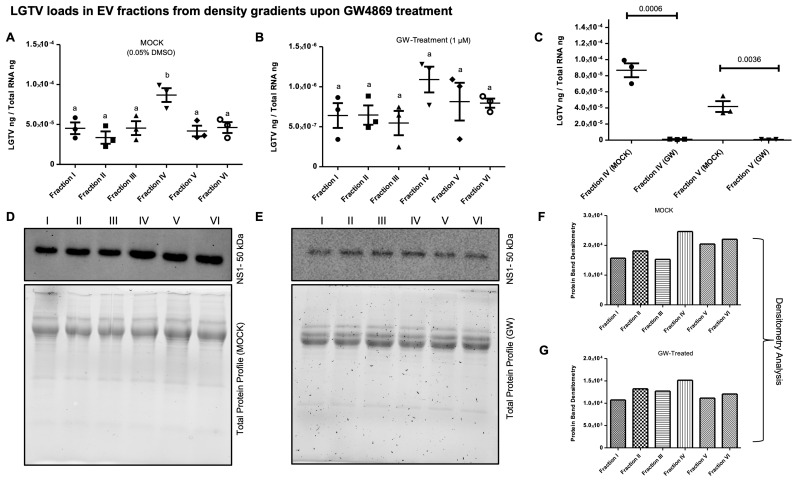
LGTV loads upon GW4869 treatment in EV fractions collected from density gradients. RT-qPCR analysis showing LGTV loads in mock-treated (0.01% DMSO) (**A**) or GW4869-treated (1 µM, for 4 h) (**B**) EV fractions (I–VI), derived from LGTV-infected-tick cells (with MOI 1, and 3 days post-infection). Both mock- and GW4869-treated groups were infected with LGTV. (**C**) Comparison of LGTV loads in fractions IV and V collected from mock- or GW4869-treated EVs derived from infected tick cells is shown. LGTV transcript levels were normalized to total RNA levels. Each closed-circle/square/triangle/inverted triangle or open circles denotes the mock- or GW4869-treated group in panels (**A**–**C**). The *p*-value less than 0.05 is considered as statistically significant, and letter b on fraction IV (in the mock-treated group) denotes significance. Immunoblotting analysis showing LGTV NS1 protein levels in mock- or GW4869-treated EVs fractions (I–VI) collected from infected tick cells (**D**,**E**). Total protein-profile gel images serve as loading controls (**D**,**E**). Densitometric analysis showing the differences in band intensities between different fractions (1–6) from LGTV-infected mock- (**F**) or GW4869-treated (**G**) EVs derived from tick cells.

**Figure 7:**
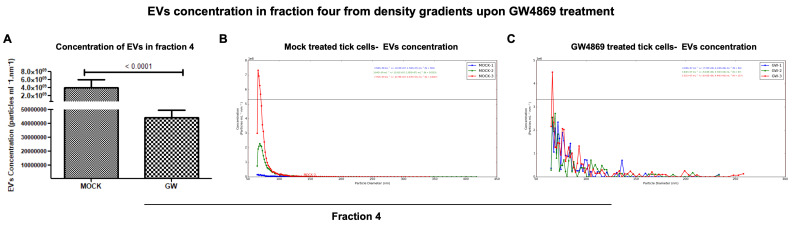
Determination of EV concentrations in fraction IV upon GW4869 treatment. Concentration of EVs in fraction IV (**A**) determined by nCS1 analysis from mock/GW4869-treated (1 µM, for 4 h) tick cells infected with LGTV is shown. Graphical representation (generated by the instrument) showing EV quantification by nCS1 analyzer in mock- (**B**) or GW4869-treated (**C**) tick cells infected with LGTV. The diameter of each particle and concentration of EVs were analyzed at the same time. Three independent replicates (in duplicates) were considered for measuring EV concentration. Both mock- and GW4869-treated groups were infected with LGTV. In panel A, *p*-value less than 0.05 is considered as statistically significant in (**A**).

**Figure 8:**
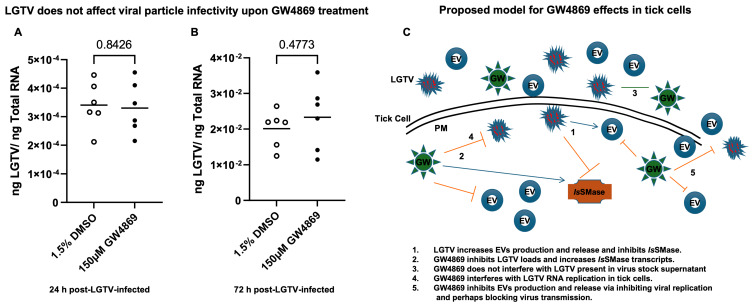
LGTV does not affect the viral particle infectivity in naïve tick cells and the model proposed for GW4869 effects in tick cells. RT-qPCR analysis showing LGTV viral loads at 24 h (**A**) or at 72 h (**B**) in naïve/uninfected tick cells that were infected via incubation with LGTV viral particles, treated with mock (1.5% DMSO, for 4 h) or with GW4869-treated (150 µM, for 4 h), virus groups. LGTV transcript levels were normalized to total RNA levels. Each open or closed circle denotes the mock-treated or GW4869-treated groups, respectively, in panels (**A**,**B**). The p-value less than 0.05 is considered as statistically significant. (**C**) LGTV induces the production of EVs and inhibits *Is*SMase sphingomyelinase. GW4869 inhibits LGTV viral replication and hampers the LGTV transmission/transport into EVs derived from infected tick cells. GW4869 reduces the EV numbers and concentrations by blocking the production and secretion of EVs. The numbers shown in the figure indicate the steps described.

**GW4869 inhibited LGTV replication correlated with a reduction in EVs released in density-gradient fractions.** RT-qPCR analysis in LGTV-infected tick cell-derived EV fractions collected from the mock-treated (0.01% of DMSO) control group revealed significantly (*p* < 0.05) higher LGTV load detection in fraction IV when compared to other fractions I-III and V, VI from the same mock group (Figure 6A). However, viral protein levels detected in LGTV-infected tick cell-derived EVs from GW4869-treated (at 1 µM) groups showed no significant (*p* > 0.05) differences between the fraction IV and other fractions from the inhibitor-treated group (Figure 6B). We noted significantly (*p* < 0.05) higher LGTV loads in all fractions from the mock-treated group when compared to the viral protein levels in GW4869-treated fractions (Figure 6A–C). Data from fractions IV and V has been shown for representation (Figure 6C). Immunoblot analysis revealed increased LGTV NS1 (non-structural) protein levels in all fractions from the mock-treated group when compared to NS1 protein levels in GW4869-treated fractions (Figure 6D,E). Total protein-profile gel images, which included all fractions from mock/GW4869-treated groups, showed no differences, and served as a loading control (Figure 6D,E). Full-length immunoblots are shown for image details (Supplementary Figure S4). Densitometric analysis showed quantitative data for significant (*p* < 0.05) differences in LGTV NS1 protein loads in all EV fractions from the mock-treated group when compared to viral NS1 protein levels in GW4869-treated EV fractions (Figure 6F,G). Furthermore, the Spectradyne nCS1^TM^ particle analyzer revealed that GW4869 treatment (at 1 µM, for 4 h) resulted in a significant (*p* < 0.05) reduction in EV concentration when compared to the mock-treated control group (Figure 7A). Measurements of EV concentration using nCS1 particle analysis revealed that results for the GW4869-treated LGTV-infected tick cell-derived EV fraction IV were significantly (*p* < 0.05) fewer in number (N = 84, 97, and 157) (Figure 7A–C). We also noted reduced concentration, with 4.04 × 10^7^ ± (7.33 × 10^6^, 4.43 × 10^6^)/mL; 3.64 × 10^7^ ± (5.84 × 10^6^, 3.74 × 10^6^)/mL, and 5.51 × 10^7^ ± (6.45 × 10^6^, 4.44 × 10^6^)/mL of EVs derived from the GW4869-treated LGTV-infected group when compared to EVs derived from mock-treated LGTV-infected tick cell-derived exosomal fraction IV (Figure 7B,C). Reduction in EV number and concentration upon GW4869 treatment directly correlated with reduced viral RNA levels in tick cell-derived EVs and in specific EVs from fraction IV. Decreased viral loads and EV numbers/concentrations in the total pool of EVs or in EVs collected as size-variable fractions further suggested that GW4869 inhibited viral replication in tick cells. In addition, we noted that mock/GW4869-treated LGTV viral particles (from laboratory viral stocks) do not affect the viral particle infectivity in naïve tick cells that were incubated either with the GW4869-treated (150 µM, for 4h) group or the mock-treated (1.5% DMSO-treated) control group. Tick cells infected with either treatment groups were collected at tested time points of 24 h (Figure 8A) or 72 h (Figure 8B) post LGTV-infection. These data show that GW4869 treatment reduced LGTV replication alone but did not affect the viral particle infectivity in naïve tick cells. 

## 4. Discussion

Given the importance of EVs and their advantages in several different therapeutics, it is essential to understand their functions. The mechanism(s) of EVs biogenesis and trafficking are very complex processes and include several molecules that are essentially involved in their production and release [10,11]. EV production, release and circulation could be blocked or inhibited with GW4869 treatment, a cell-permeable, selective inhibitor of N-SMase (neutral sphingomyelinase), which is a pharmacological agent that blocks the production and release of EVs [1,2,3,5]. Our previous studies have shown that tick and mosquito EVs facilitate transmission of flaviviruses between the medically important vectors to the vertebrate host [4,7,8,16,20]. These studies revealed that GW4869, reduces the viral RNA levels and efficiency of several flaviviruses (such as Langat virus, LGTV, Dengue, ZIKA and West Nile viruses) transmission (via infectious EVs) to the host cells. Inhibition of viral RNA levels by GW4869 treatment was noted in tick, mosquito, and several of the mammalian cells, which included human skin keratinocytes (HaCaT cells), human blood endothelial cells (HUVEC), mouse N2a cells, and the primary cultures of murine cortical neurons [4,7,8,9,20]. However, the mechanism for this reduction in flaviviral RNA levels by GW4869 treatment was unknown.

To understand how GW4869 affects the viral RNA levels in host cells, we hypothesized that GW4869 perhaps affects flaviviruses by direct binding or by interfering with the viral RNA or replication process. In our previous studies, we pre-treated tick/mosquito/mammalian cells with GW4869 (for 4h), followed by infection with the respective flaviviruses. Our current study, with similar method of treatment, showed that GW4869 (at tested doses) neither showed morphological changes nor had any cytopathic effects in tick cells (as revealed by the LDH-Glo cytotoxic assay). Also, the MTT assay showed no differences in cell viability of tick cells upon GW4869 treatments (at 50 and 150 µM) when compared to the mock-treated group, thus further suggesting no cell death. We then tested whether GW4869 interferes with LGTV RNA present in the laboratory viral stocks. We noted that GW4869 treatment did not directly affect LGTV RNA levels in the undiluted or diluted laboratory virus stock suspensions. These data suggested that GW4869 perhaps affects the LGTV replication process inside the host cells, and not by directly interfering with the virus in suspension. We believe that GW4869 does not directly affect viral RNA in LGTV virus stocks, but it may affect the virus infectivity (as shown by the virus dilution assays) and/or build-up, or accumulation of sphingomyelin lipids required for the viral replication process at the inner leaflets/lipid rafts of tick cells. Our immediate future research will address whether GW4869 treatment affects the viral RNA genome content that could allow the lack of direct interaction. The virus dilution assay performed with GW4869-treated (both 1 and 150 µM) tick cells showed that only 1 µM dose of the inhibitor and 3 days post-infection with the virus is sufficient to decrease LGTV replication in tick cells. A higher dose of GW4869 (150 µM) and increased time of 6 days post-incubation with the virus further reduced viral infectivity or the infectious dose of LGTV. This data suggests that GW4869 is a potential therapeutic agent for controlling tick-borne flaviviruses. The current observation, that GW4869 treatment reduced LGTV replication in tick cells and decreased the transmitted viral RNA levels in EVs and EVs fractions derived from tick cells, further corroborated our previous reports [4,7,8,9]. Our data showed that GW4869-mediated treatment reduced LGTV replication in tick cells and affected the viral transmission/transport via tick-cell derived EVs to naïve recipient mammalian cells (human-skin keratinocytes or blood endothelial cells) [4,8]. Furthermore, increased expression of *Is*SMase upon GW4869 treatment confirmed our previous study findings [4] that LGTV inhibits the tick sphingomyelinase (*Is*SMase- *Ixodes scapularis*, black-legged tick) gene transcript. This sphingomyelinase is a specific ortholog of highly venomous spider (*Loxosceles similis*) sphingomyelinase, SMase D. *Is*SMase is not an ortholog of mammalian N-SMase2/SMPD3, and we consider this tick sphingomyelinase as a unique molecule. We have reported that treatment of tick cells with GW4869 reduces LGTV loads but increases *Is*SMase levels. We have shown [4] that LGTV infection results in accumulation of sphingolipids such as sphingomyelin (by inhibiting both the sphingomyelinase activity and *Is*SMase levels upon LGTV infection). However, treatment with GW4869 reverses all these effects and increases *Is*SMase levels and the sphingomyelinase activity, by reducing the levels of sphingomyelin lipid [4]. We assume that increased expression of *Is*SMase upon GW4869 treatment leads to a reduction in sphingomyelin lipid levels (by converting it to ceramides and phosphocholine). The mechanism of GW4869 on *Is*SMase is still unknown, and our immediate future studies will address these mechanistic details. We believe that *Is*SMase could be an important antiviral candidate in ticks.

EVs have been given a high prominence as therapeutics for curing several diseases, including in cancer therapy [25,26,27,28,29,30,31,32,33,34,35,36,37,38]. In addition to this evidence, our current study shows, for the first time, that GW4869 treatment (1 and 150 µM) significantly reduced the viral RNA levels in EVs and affected the secretion/release of EVs from LGTV-infected tick cells. These data, showing a reduction in EV concentrations upon GW4869 treatment, suggested that a decrease in LGTV replication and transmission efficiency (via infectious EVs) is not only due to reduced viral replication in tick cells, but also due to a dramatic decrease in total EV concentration, which hampers virus transmission and dissemination to naïve recipient cells. Our current finding, that GW4869 treatment (1 and 150 µM) reduced EV release from tick cells (in vitro), further promoted us to investigate the EV heterogenous population. Therefore, we analyzed the EV fractions (I–VI) collected by density gradient with size variations to correlate the reduced LGTV loads with a decrease in EV concentration. Our data revealed that EVs contain higher viral RNA or protein levels in fractions IV and V, which corroborated with our previous study [8]. The current study showed that GW4869 treatment greatly affected LGTV loads from those fractions IV and V, and this reduction correlated with decreased EV concentrations. Our data also suggested that GW4869 treatment of tick cells affects EV secretion and release, perhaps by reducing the viral replication. The reduction in EVs from fractions IV correlated with the decrease in LGTV replication, thus suggesting that GW4869 specifically targets EVs of small sizes, which contain a large amount of the viral RNA and proteins. Our study suggests that GW4869 targets EV release by decreasing the viral replication process and transmission to naïve recipient cells. In summary, some of the important findings from this study are the following: (a) GW4869 is not toxic at the highest tested doses (of 50 and 150 µM) in tick cells; (b) GW4869 does not interfere with LGTV viral RNA; (c) GW4869 consistently interferes with LGTV replication in tick cells (at both tested doses of 1 and 150 µM); (d) GW4869 reduces the infectious dose of LGTV (viral titers determined by the tissue culture infectious dose, TCID_50_, were affected in the GW4869 group and at both tested doses of 1 and 150 µM); (e) GW4869 reduces LGTV loads in both tick cells and in EVs (and at both tested doses of 1 and 150 µM); and (f) the current work presents how LGTV loads are not just reduced in EVs, but how GW4869 significantly affects the EV concentrations in pooled EV preparations or from density-gradient preparations of EVs collected as different fractions (and, specifically, from fraction IV). Our data suggested that GW4869 treatment reduced LGTV replication but does not specifically affect its viral particle ability to infect naïve tick cells. A summarized model is proposed for these findings (Figure 8C). Overall, this study suggests that GW4869 is a potential therapeutic in controlling tick-borne diseases.

## Data Availability

The original data presented in the study are openly available in this open access publication.

## Supplementary Materials

The following supporting information can be downloaded at: https://www.mdpi.com/article/10.3390/v17070969/s1. **Figure S1:** Higher magnification or insets of tick cells shown in Figure 1C. For better visualization, insets are shown for images from uninfected tick cells treated with mock (1.5% DMSO) or GW4869 (at two doses of 50 and 150 µM) treatments.; **Figure S2:** RNaseA treatment on GW4869-treated free LGTV present in laboratory viral stock suspensions. (**A**) Schematics showing experimental plan for the incubation of LGTV laboratory viral stocks (undiluted suspension with 1 × 1e7 pfu/ml) with GW4869 (1 µM) or mock (0.01% DMSO) control (for 4 h at 37 °C incubation) followed by digestion with RNaseA. (**B**) RT-qPCR analysis showing LGTV viral loads in mock/GW4869-treated undiluted virus stocks. LGTV prM transcript levels were normalized to total viral RNA levels. Open circles denote mock-treated group, and solid circles represent GW4869-treated group. Each circle represents one experimental replicate (100 µl) of undiluted virus suspension and n = 6 replicates run in duplicates. The *p*-value less than 0.05 is considered statistically significant.; **Figure S3:** Determination of LGTV-infectious dose in GW4869-treated tick cells. Fluorescent images collected from viral dilution assay (from three individual microscopic fields) of LGTV-infected tick cells treated with either mock (0.01% DMSO) control (**A**) or GW4869 (1 µM) inhibitor (**B**) from dilutions of 10^−2^, 10^−3^ and 10^−4^ are shown. Differences in viral titers determined from mock or GW4869-treated tick cells are shown as percentages (on right side of each image group). Tick cell images were obtained using Cytation7 imager and scale bar of 200 µm is shown for each image.; **Figure S4:** Full size immunoblots images of fractions. Immunoblots represented in Figure 6 (for LGTV NS1 protein detection) are shown again in their full sizes. M indicates marker and Roman numerals (1–VI) indicates 6 independent fractions (I–VI) obtained from the density gradient preparations of mock (0.01% DMSO) or GW4869 (1 µM)-treated groups.
