# Supplementary material for: Pharmacological Agent GW4869 Inhibits Tick-Borne Langat Virus Replication to Affect Extracellular Vesicles Secretion
Source: Viruses. 2025 Jul 10;17(7):969. doi: 10.3390/v17070969 (PMC12298192; doi:10.3390/v17070969)
Supplement: Supplementary file 1 [file viruses-17-00969-s001.zip › viruses-3598567-supplementary.pdf]

SUPPLEMNETAL FIGURES AND LEGENDS

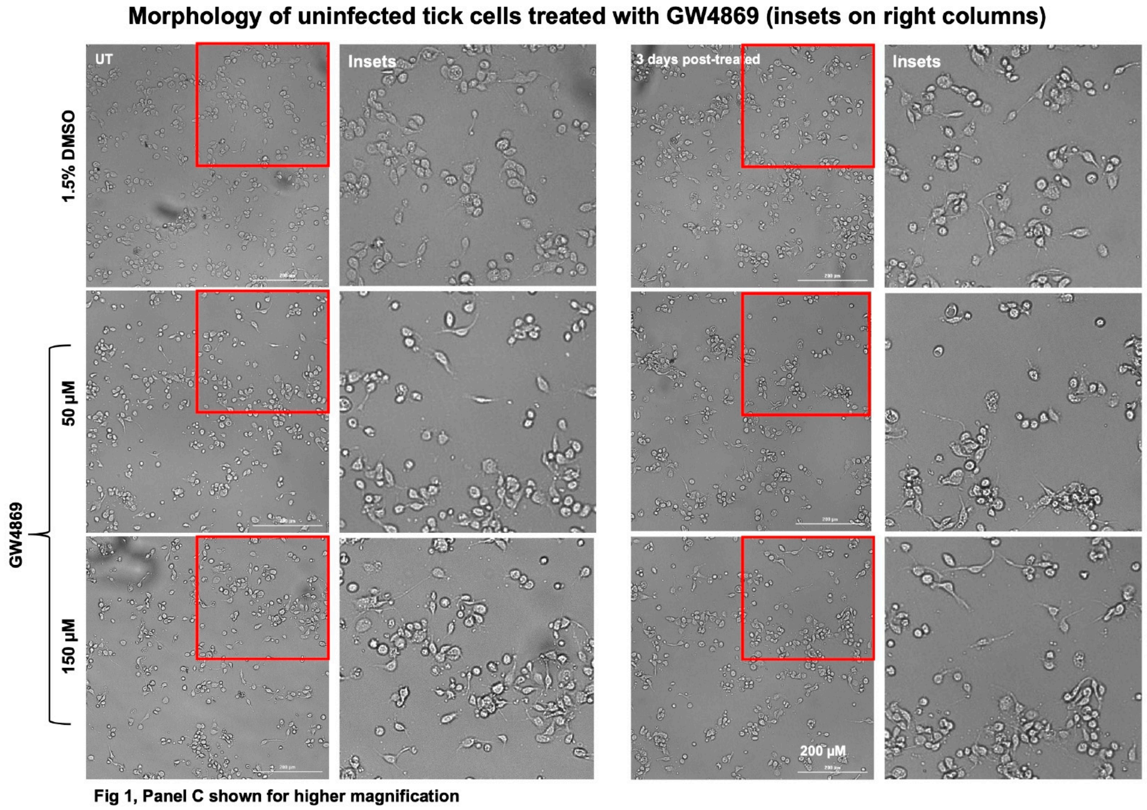

Supplementary Figure S1. Higher magnification or insets of tick cells shown in Figure 1C. For better visualization, insets are shown for images from uninfected tick cells treated with mock (1.5% DMSO) or GW4869 (at two doses of 50 and 150 µM) treatments.

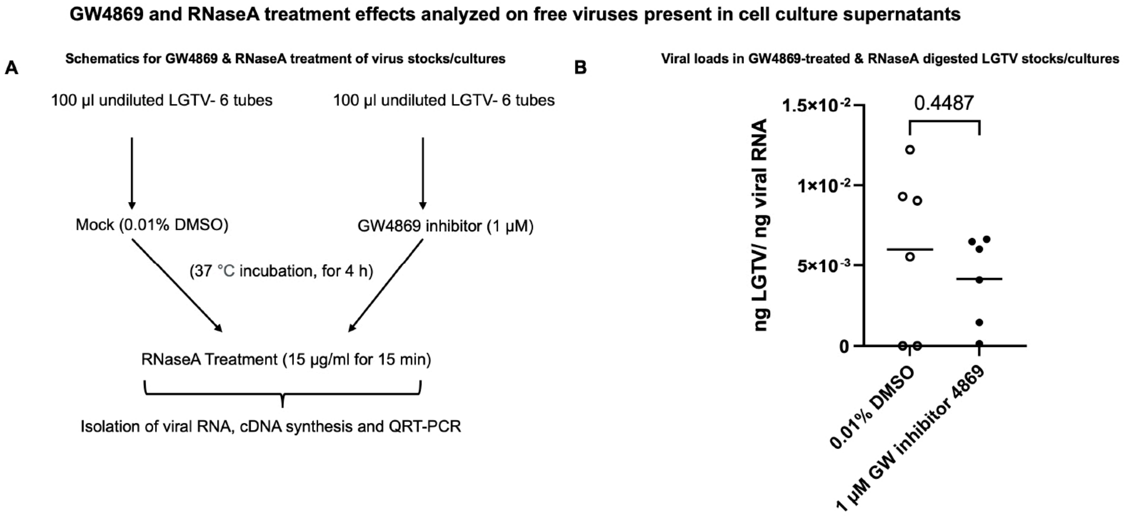

Supplementary Figure S2. RNaseA treatment on GW4869-treated free LGTV present in laboratory viral stock suspensions. (A) Schematics showing experimental plan for the incubation of LGTV laboratory viral stocks (undiluted suspension with 1 x 1e7 pfu/ml) with GW4869 (1 µM) or mock (0.01% DMSO) control (for 4 h at 37 ºC incubation) followed by digestion with RNaseA. **(B)** RT-qPCR analysis showing LGTV viral loads in mock/GW4869-treated undiluted virus stocks. LGTV prM transcript levels were normalized to total viral RNA levels. Open circles denote mock-treated group, and solid circles represent GW4869-treated group. Each circle represents one experimental replicate (100 µl) of undiluted virus suspension and n=6 replicates run in duplicates. The p-value less than 0.05 is considered statistically significant.

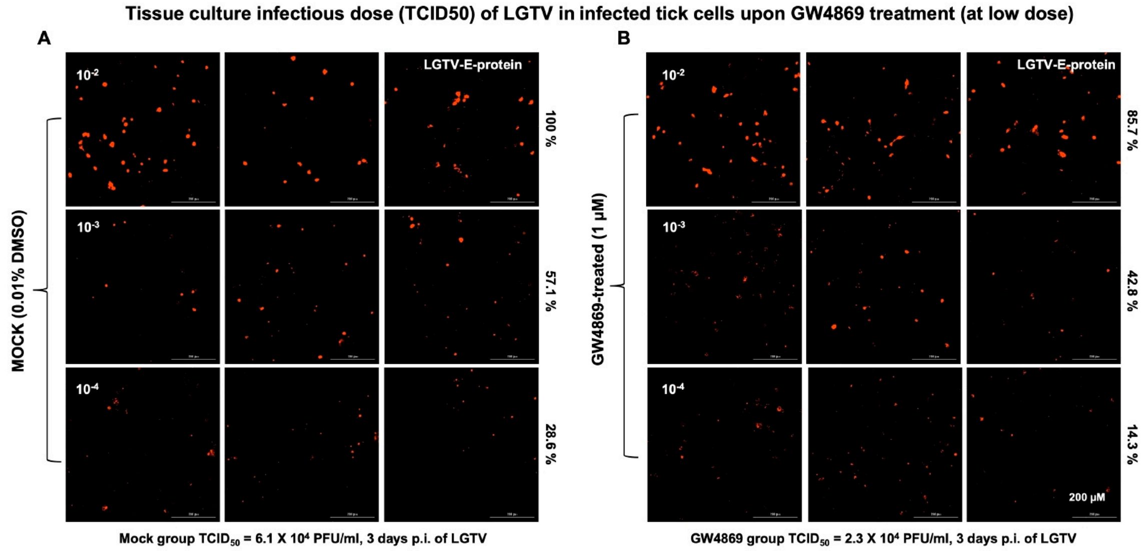

Supplementary Figure S3. Determination of LGTV-infectious dose in GW4869-treated tick cells. Fluorescent images collected from viral dilution assay (from three individual microscopic fields) of LGTV-infected tick cells treated with either mock (0.01% DMSO) control **(A)** or GW4869 (1 µM) inhibitor **(B)** from dilutions of 10^{-2}, 10^{-3} and 10^{-4} are shown. Differences in viral titers determined from mock or GW4869-treated tick cells are shown as percentages (on right side of each image group). Tick cell images were obtained using Cytation7 imager and scale bar of 200 µm is shown for each image.

Full-size images of immunoblots shown in Figure 6

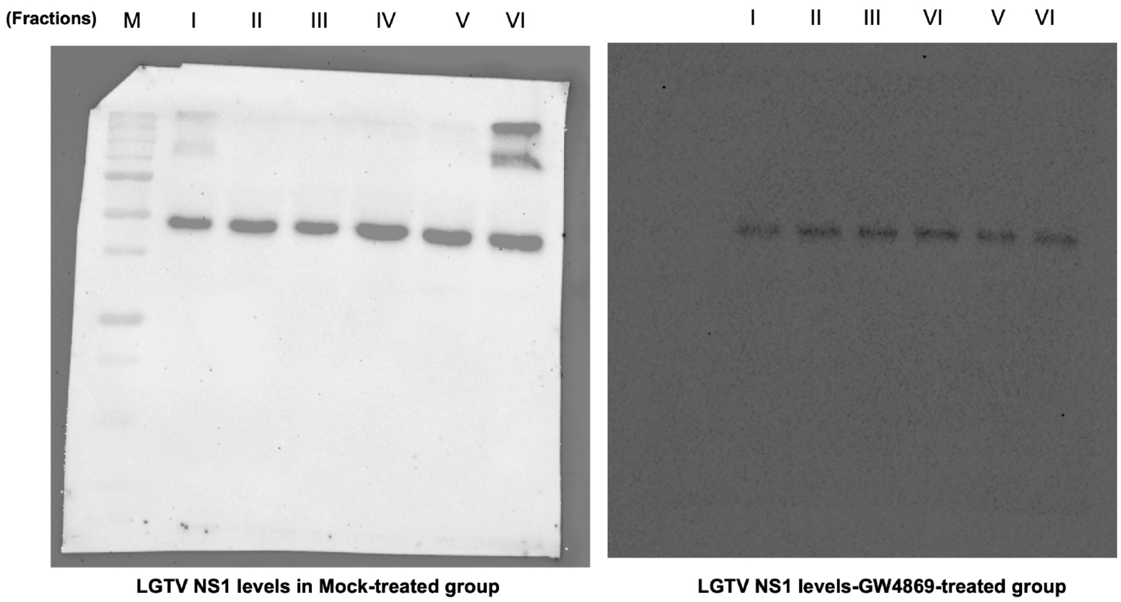

Supplementary Figure S4. Full size immunoblots images of fractions. Immunoblots represented in Figure 6 (for LGTV NS1 protein detection) are shown again in their full sizes. M indicates marker and Roman numerals (1-VI) indicates 6 independent fractions (I-VI) obtained from the density gradient preparations of mock (0.01% DMSO) or GW4869 (1 µM)-treated groups.
